# A Study of the Recent Trends of Immunology: Key Challenges, Domains, Applications, Datasets, and Future Directions

**DOI:** 10.3390/s21237786

**Published:** 2021-11-23

**Authors:** Sharnil Pandya, Aanchal Thakur, Santosh Saxena, Nandita Jassal, Chirag Patel, Kirit Modi, Pooja Shah, Rahul Joshi, Sudhanshu Gonge, Kalyani Kadam, Prachi Kadam

**Affiliations:** 1 Symbiosis Institute of Technology, Symbiosis International (Deemed) University, Pune 412115, India; aanchal.thakur.btech2018@sitpune.edu.in (A.T.); saxena.santosh.btech2018@sitpune.edu.in (S.S.); nandita.jassal.btech2018@sitpune.edu.in (N.J.); rahulj@sitpune.edu.in (R.J.); sudhanshu.gonge@sitpune.edu.in (S.G.); kalyanik@sitpune.edu.in (K.K.); prachi.kadam@sitpune.edu.in (P.K.); 2 Computer Science & Engineering, Devang Patel Institute of Advance Technology and Research, Changa 388421, India; chirag453@gmail.com; 3 Sankalchand Patel College of Engineering, Sankalchand Patel University, Visnagar 384315, India; kjmodi.fet@spu.ac.in; 4 Information Technology Department, Gandhinagar Institute of Technology, Ahmedabad 382010, India; pooja.shah@git.org.in

**Keywords:** immunology, AI in healthcare, machine learning, deep learning, diagnosis

## Abstract

The human immune system is very complex. Understanding it traditionally required specialized knowledge and expertise along with years of study. However, in recent times, the introduction of technologies such as AIoMT (Artificial Intelligence of Medical Things), genetic intelligence algorithms, smart immunological methodologies, etc., has made this process easier. These technologies can observe relations and patterns that humans do and recognize patterns that are unobservable by humans. Furthermore, these technologies have also enabled us to understand better the different types of cells in the immune system, their structures, their importance, and their impact on our immunity, particularly in the case of debilitating diseases such as cancer. The undertaken study explores the AI methodologies currently in the field of immunology. The initial part of this study explains the integration of AI in healthcare and how it has changed the face of the medical industry. It also details the current applications of AI in the different healthcare domains and the key challenges faced when trying to integrate AI with healthcare, along with the recent developments and contributions in this field by other researchers. The core part of this study is focused on exploring the most common classifications of health diseases, immunology, and its key subdomains. The later part of the study presents a statistical analysis of the contributions in AI in the different domains of immunology and an in-depth review of the machine learning and deep learning methodologies and algorithms that can and have been applied in the field of immunology. We have also analyzed a list of machine learning and deep learning datasets about the different subdomains of immunology. Finally, in the end, the presented study discusses the future research directions in the field of AI in immunology and provides some possible solutions for the same.

## 1. Introduction

**Figure 1:**
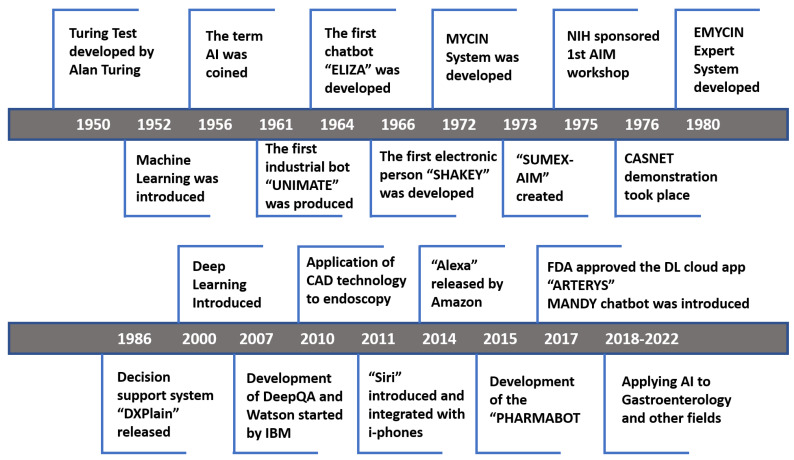
A Year-wise Evolution of AI in Healthcare (1950–present).

Healthcare is formally defined as the maintenance, restoration, and improvement of an individual’s physical, mental, and emotional health. It involves the prevention, detection, and treatment of diseases and injuries usually provided by trained specialists and others in the medical field [1]. The current scenario encompasses the entire sphere of services, aspects, and devices that can care for a person’s health [2]. The latest trends in the field of healthcare, in particular in the past two years, are indicative of a major shift in how healthcare services are provided to the general public. The style and approach of healthcare facilities are moving towards outpatient rather than inpatient facilities. Some of the best examples of this trend show the rise in ambulatory surgery centres, imaging facilities, and retail clinics. This wave of change is riding on the back of technological enhancements and breakthroughs in technologies in the healthcare industry. It includes the incorporation of data warehousing and big data concepts into the standard data collection procedures of imaging centres and emergency departments. Cloud computing, sophisticated diagnostic tools, telemedicine systems, and innovative mobile technologies are some of the most upcoming trends in the healthcare field [3]. Consolidation or merging of healthcare entities is, unfortunately, a trend that does not seem to be stopping soon and will lead to an increase in the healthcare prices and dominance of a few large and prominent players in the healthcare markets in the coming years. This consolidation is believed to be a direct result of the impact of the Affordable Care Act [4]. Thirdly, with the increasing advent of big data and other technologies in healthcare, such as AI/ML, there is a higher focus on ensuring patient data confidentiality. Efforts such as the California Consumer Protection Act (CCPA), enacted in 2020, aim to promote healthcare data security [5]. Finally, the emergence of even more technologies, including the rapid integration of AI/ML into the field of telehealth, is leading to the rapid consumerization of healthcare services. The current COVID 19 pandemic has seen an upsurge in telehealth and remote healthcare services as the social distancing norms are being enforced globally. Thus, now more than ever, the application and scope of artificial intelligence in healthcare is rapidly expanding. One of the biggest advantages of using AI in the field of healthcare is that it can lead to improvements in patient experiences and increased support in the field of outcomes and healthcare services. This process further leads to enhancements in the efficiency of the entire healthcare delivery process, making it possible to provide good quality healthcare services to more people. It also increases automation in many tedious procedures such as data collection and image analysis, thus reducing the workload on the healthcare practitioners, which allows them to devote more one-on-one time to the patients in their care [6]. Furthermore, human beings are mortal. Not just because of the process of ageing, but we are also highly susceptible to various diseases. They can be caused due to various reasons. For example, they may be caused due to genetic deformities, such as the lack of a particular DNA strand, or external pathogens such as viruses, bacteria, etc. Identifying these diseases in the correct time frame and then treating them and preventing their spread in the case of infectious diseases is vital. While doctors do an exceptional job of performing most of these tasks, there are certain areas where using AI/ML/DL augments their performance. These algorithms can figure out trends and similarities in the data values available faster than humans and even be able to identify anomalies that human beings cannot see or understand. Therefore, AI is finding an application in almost all fields of medicine to improve the detection, treatment, and prevention of diseases. Table 1 represents the list of used terminologies. Figure 1 shows the year-wise evolution of AI in healthcare technologies from 1950 to the present.

AI is not a new field; it has developed over the years, facings times of high boom and no advancements, also called the AI winters. The first time artificial intelligence was introduced was when Alan Turing developed the Turing Test in 1950. It was originally called the Imitation Game. It was a means of testing a machine’s capability to showcase intelligent behaviour greater than or at par with that of a human being’s. In 1952, machine learning was introduced, and finally, the term Artificial Intelligence was coined in 1956 [7]. From then on, the potential of Artificial Intelligence in healthcare was also recognised. In 1964, the first chatbot ELIZA was developed. This chatbot was an extended test case for the Turing Test, and it was aimed at fooling its users into thinking that they were having a conversation with a real human being. In 1972, MYCIN, a backward chaining expert, was developed by applying artificial intelligence concepts to diagnosing patients based on their symptoms and medical test results. It could identify the bacteria causing infections like meningitis and was even able to recommend medication (antibiotics) in the correct doses calculated as per the patient’s body [8]. In 1973, the SUMEX-AIM network was created. It was a nationally-shared computing resource whose sole aim was to design artificial intelligence applications for the biomedical sciences, and in 1975, the first AIM Workshop was sponsored by NIH. CASNET/Glaucoma was a casual-associational network model used to describe the disease processes, particularly in the case of glaucoma [9]. A clinical decision support system (CDSS) named “DXplain” was introduced in 1986. It assisted the clinicians by using the inputs of patent data provided to generate stratified diagnoses, and evidential support and probable follow-ups aid the clinicians with arriving at the definitive diagnosis. Furthermore, it also acts as a resource database for doctors. With the introduction of deep learning in 2000, IBM soon developed its DeepQA and Watson systems for evidence-based reasoning and deep content analysis in the year 2007. The Watson system was developed as part of the DeepQA project to act as a question-answering computer that could interact with the users and respond to questions posed in the Natural Language [10]. What followed next was a decade of the sudden boom in the applications of AI and AI-based assistants to make them available to the general public on a day-to-day basis like Alexa and Siri. In 2015, PHARMABOT, the pediatric Generic Medicine Consultant Chatbot, was introduced, which suggested generic medicines for children, and in 2017, the MANDY chatbot was introduced [11]. Cloud technologies were leveraged along with deep learning, and since then, the focus has been on applying AI to all possible healthcare fields in any way possible to aid the doctors.

**Figure 2:**
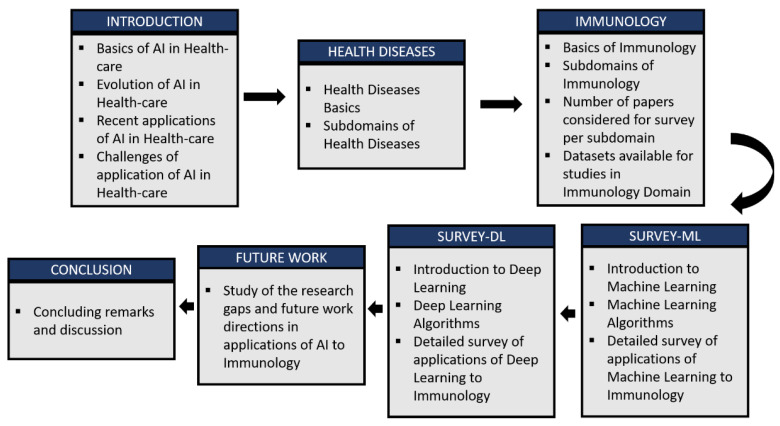
The roadmap representation of the undertaken study.

Roadmap of the undertaken study: The roadmap of the proposed review has been represented in Figure 2. In the undertaken study, a systematic literature survey has been conducted focusing on AI in healthcare, applications of machine learning techniques to immunology, and deep learning applications to the different subdomains of immunology. In this review, we have referred to reputed journals and conferences of reputed publishers, such as IEEE, Springer, Elsevier, Willey, and many more. In addition, Scopus and Web of Science search engines are used for reliable results. For searching relevant articles, keywords used are healthcare AI, immunology, deep learning, machine learning, etc. The rest of the paper is organized as follows. Section 4 provides a discussion on what are health diseases and their subcategorizations. Section 5 presents a detailed discussion of immunology as a domain and the different subdomains which come under it, including autoimmune diseases, immunodeficiency diseases, development of vaccines, etc. Section 6 and Section 7 described the detailed review of the state of the arts and most recent applications of machine learning and deep learning, respectively, in immunology. Finally, future directions for research are provided in Section 8 and are followed by the closing remarks in Section 9.

## 2. Recent AI Contributions in Healthcare

The combination of healthcare and technology has been proven to be the best to date. Using top-level technologies to build strong and reliable healthcare solutions has been one of the major achievements. AI is being widely used in healthcare to prevent, diagnose, and treat diseases and act as a helping hand to medical professionals [4]. For example, computer vision is widely used in medical imaging. It can thoroughly understand features and patterns hidden in the images leading to a better and more reliable diagnosis of possible cavities, tumors, and much more. Studies also indicate that deep learning algorithms perform remarkably in medical imaging. In one of the studies, researchers concluded that Convolutional Neural Networks performed better than deep neural networks and stacked autoencoders to diagnose lung cancer. Similarly, many machine learning techniques are also used for cancer detection; the work revolves around using ML/DL algorithms to detect tumors and classify them into malignant or benign groups for further medical procedures [12,13].

AI is widely used in drug discovery and development, largely to find new uses of existing drugs, optimize their effectiveness, and much more [14,15,16,17,18]. Research has been done using AI models to detect adverse event under-reporting to ensure patient safety and integrity of the generated data [19]. Apart from the prevention, diagnosis, and treatment part, AI has been very helpful in assisting medical staff to ease the workload. With chatbots, early communication with the patient has been significantly effective to understand symptoms better. Many predictive AI models are used to understand the patients and risk levels better in order to decide which patient needs utmost care and facilities at the earliest. For surgeries, robotic-assisted surgeries are also coming up for better precision. Many AI tools help doctors check real-time details of the patients during critical surgeries [20,21,22,23].

### 2.1. Application of AI in Various Healthcare Domains

**Figure 3:**
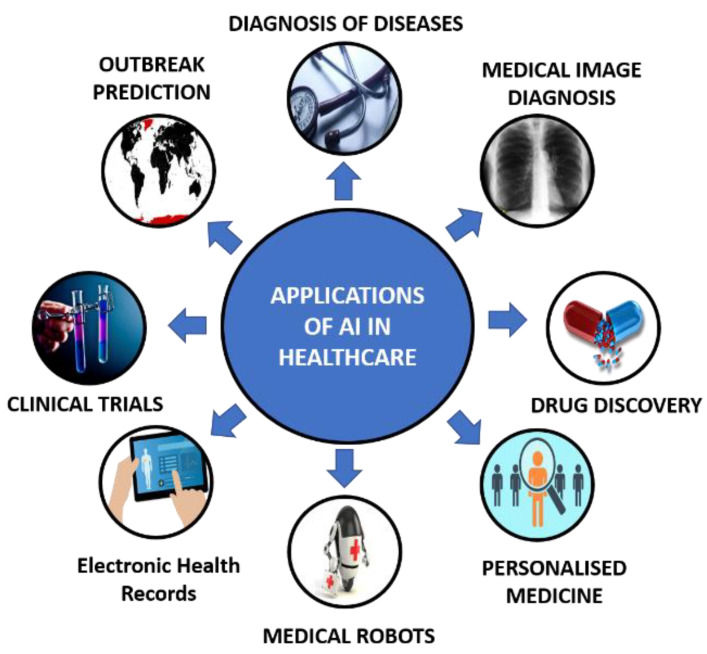
A representation of various applications of AI in healthcare.

For the longest period, the healthcare field has resisted introducing machines and has remained a human-dominated area. A big reason for this was that most machines were not capable of matching the depth of knowledge, delicateness, precision, and expertise that doctors had until recent times. However, this all started to change when machines became capable of performing feats that had seemed virtually impossible until then. X-ray machines, CT Scanners, etc., soon became the norm in every medical facility. Still, it was considered that machines would never be able to beat humans. However, with the advent of artificial intelligence and machine learning, it was realized that machines could become “intelligent” to some extent. Furthermore, studies have shown that a collaborative work effort between AI and doctors has led to improved performance in all the areas where this collaboration happened. Figure 3 represents various applications of AI in healthcare.

#### 2.1.1. Diagnosis and Treatment of Diseases

**Figure 4:**
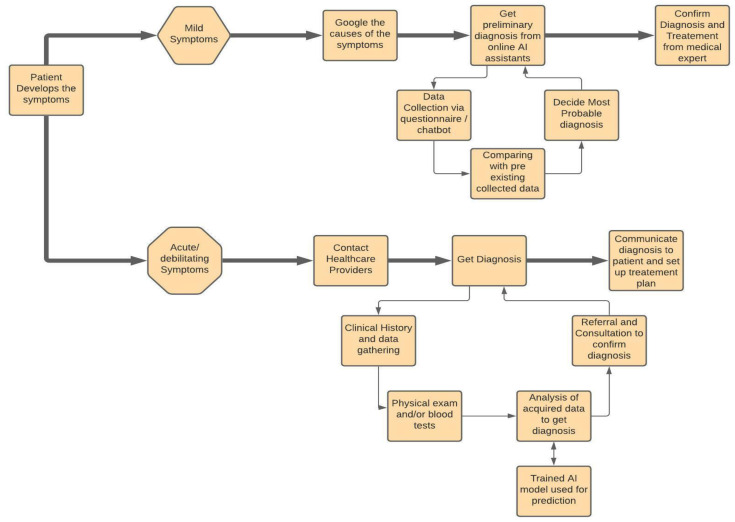
The Process Flow Representation of Diagnosis of Diseases using AI.

The interest in using AI/ML in the health industry and diagnosing and treating diseases started as early as the 1970’s. Machine learning algorithms work on the base concept of computational statistics. In short, once the data are provided to the algorithm, it learns and explores the connections within the data points and tries to reveal the complex patterns between the data that may not even seem understandable to a human. This property enables it to be used in the detection of diseases that are not easy to identify in their early stages, but which can be disastrous if left to progress to their later stages. One such example is cancer. It is exceedingly difficult for the human eye to spot the beginning signs of a tumor or detect whether they are malignant or benign. However, machine learning can easily find these early indicative signs with high accuracy. IBM’s Watson Genomics [24] is one of the leading examples of how genome-based tumor sequencing integrated with cognitive computing can help quickly diagnose [12]. Another example of this application is the machine learning algorithm that the researchers developed at MD Anderson to predict acute toxicities in patients who suffer from head and neck cancers and are receiving radiation therapy [13]. The biopharma giant Berg is also using the concepts of AI in the field of therapeutic treatments in areas such as oncology. Fellow researchers are conducting research work to develop systems that can be integrated into routine clinical conditions, which will provide quick diagnosis and treatment to all patients [14]. It is important to note that AI cannot replace the opinions of a professional medical care expert; however, it can be used to corroborate the findings of these professionals and make the diagnosis that much more confirmed. Figure 4 depicts the process flow of diagnosis of treatment of diseases using AI.

#### 2.1.2. Medical Image Diagnosis

**Figure 5:**
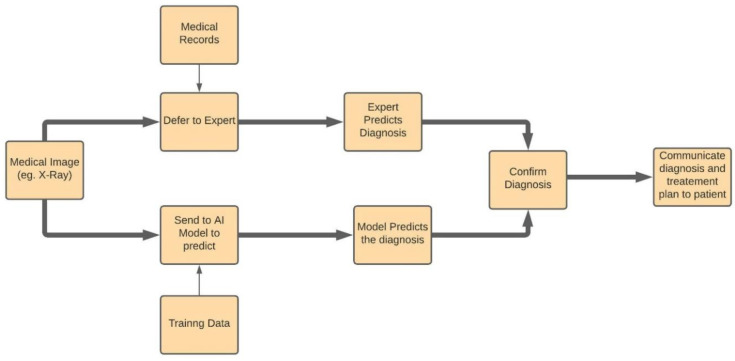
The Process Flow Representation of Medical Image Diagnosis using AI.

AI-based Computer Vision methodologies allow computers to interpret and understand the visual world, especially in healthcare [15]. Such methodologies can identify and classify objects from various visual inputs such as images and videos from cameras, etc. This technology, once introduced, managed to take the medical world by storm because of its potential applications, in particular in the field of radiology. It can detect and analyze the images’ patterns such as radiographs, PET, MRI, and CT scans. These machine learning algorithms have been shown to perform at accuracy on the same scale as experienced radiologists. For example, the Google machine learning applications in healthcare that have been trained to detect breast cancer [16] have accuracies as high as 89 percent. In addition, Microsoft has developed the InnerEye initiative, whose purpose is to work on image diagnostic tools for image analysis. Medical imaging is also one of the fields in medicine where large datasets are readily available. Therefore, medical imaging methodologies assist in developing very accurate machine learning algorithms. Figure 5 represents the process flow of medical image diagnosis.

#### 2.1.3. Drug Discovery and Manufacturing

**Figure 6:**
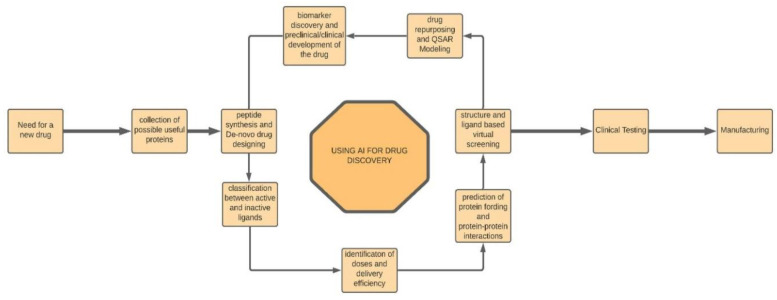
The Process Flow Representation of Drug Discovery using AI.

Long gone are the times when the discovery and development of drugs such as vaccines, etc., were considered very long and extremely hazardous processes. With the introduction of machine learning algorithms and AI robots, the process has become much quicker and safer as the machine learning algorithms handle the initial research grunt work, and other heavyweight processes such as identification of predictive biomarkers, target validation, etc., are also easily achieved using different machine learning models [17]. They can also analyze and then predict alternatives to the current paths followed for therapy, especially in multifactorial diseases or even create individual responses to the therapeutic drugs, such as cancer patients [18]. To find the new drugs, you need to, for the most part, forget the rules and look at the clues and patterns that data and previous research provide. Thus, unsupervised learning algorithms are used in this field. One of the novel frameworks for the same is the Seq2seq Fingerprint, a robust model that uses unsupervised learning for drug discovery [25]. Another example of drug discovery using AI is the Project Hanover of Microsoft [26]. It has even helped develop technologies for the treatment and personalizing of drug combinations for acute myeloid leukaemia and certain types of cancers. Figure 6 represents the process flow of drug discovery and manufacturing using AI. 

#### 2.1.4. Personalized Medicine

**Figure 7:**
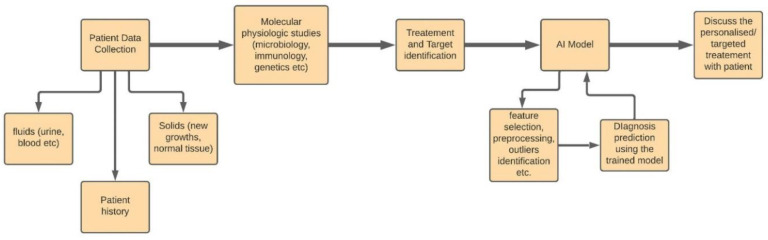
The Process Flow Representation of Personalized Medicine.

Each patient is unique, and there are many diseases for which the treatments have to be particularly tailored to suit every patient. This can be as simple as suggesting the best multivitamins to a person with low immunity or as serious as the doses and types of drugs needed for acute diseases. Furthermore, with the increasing amount of data available, especially in oncology, Electronic Health Records (EHRs) are now being designed to manage and correlate the different patient profiles by using predictive models such as SVM, ANNs, etc. [27]. The machine learning algorithms can easily sort through and learn from the vast range of treatments available and combine them with the patient’s genetics and medical history to suggest the best possible treatment for them. IBM Watson’s Oncology [28] is an example of patient history being analyzed by machine learning algorithms to generate multiple courses of action. A doctor can then review and select the most suitable one. This makes the entire process quicker and reliable. Figure 7 represents the process flow of applying AI in personalized medicine.

#### 2.1.5. Physical Robots

Physical robots are one of the most fantasized and well-documented applications of AI. More than that, statistics show that as many as 200,000 industrial robots are manufactured and used worldwide [29]. Not only do they perform the pre-defined physical labour tasks such as those on an assembly line, but they have also evolved further due to the addition of AI algorithms into their Operating Systems. They can now help in performing intelligent tasks too. The most well-known examples are the surgical robots among those in the healthcare industry. These help the surgeons perform their tasks in a more optimized manner and help make a lot of the surgeries and procedures minimally invasive. They have revolutionized the areas of gynecologic, prostate, and even head and neck surgeries. 

#### 2.1.6. Administrative Tasks and Smart Records Management

A large portion of the time of medical practitioners is usually spent on filing records and other administrative tasks. Moreover, there is a high chance of manual error in the filing systems, which could severely affect the credibility and integrity of the records. Machine learning has assisted in reducing data processing load using automation. Some of the most common technologies used for this purpose include machine learning-based OCR recognition and document classification. Google cloud’s Vision API [19] and the handwriting recognition technology of MATLAB are two examples of this particular application. MIT has also worked extensively and is nearing the cutting edge of developing intelligent health record systems. Moreover, these systems can also be modified to handle all sorts of data, including images. They can be further enhanced and integrated with other technologies such as image processing to develop systems that could even fill in a patient’s medical history based on digital copies of their previous prescriptions, analyze them, and predict a preliminary course of action for the patients [30].

#### 2.1.7. AI in Clinical Trials

**Figure 8:**
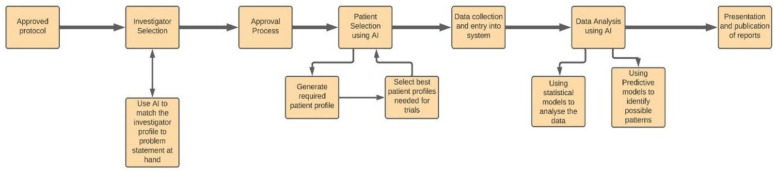
The Process Flow Representation of AI in Clinical Trials.

Machine learning can be used to identify and select potential candidates for trials based on predictive technologies. Such methodologies can assist in reporting any adverse events that occur during a clinical trial instead of doing these audits and checks manually. Machine learning can also help in the assimilation and understanding of the results from the clinical trials using clustering or classification algorithms depending upon the needs. Moreover, they can also help in drawing a pool from a wide variety of data points. Machine learning further helps in real-time monitoring of the patient’s data and other statistical tasks like deciding on the sample size, creating well-organized records, etc. Furthermore, it can also help in the accumulation of crowdsourced data. Medtronics and IBM partnered together to perform this very task for the particular case of consolidating diabetes and insulin datasets in real-time. Moreover, Apple’s ResearchKit provides users with access to applications that use machine learning based Facial Recognition to treat diseases like Asperger’s and Parkinson’s. These advancements in data collection will lead to the development of more sophisticated devices and algorithms, which will further improve the applications of AI in healthcare [31]. Figure 8 represents the process flow of AI in clinical trials.

#### 2.1.8. Predicting Outbreaks

This particular application has seen a lot of use in the recent COVID-19 times wherein AI was used to predict the extent and spread of the virus [32]. Real-time data collected globally through various mediums like social media, satellites, and even newspapers were analyzed and studied to predict the well-known curve of the spread of the coronavirus. Such methodologies can act as a good indicator and warning to all the countries to prepare themselves to get the required infrastructure to deal with the outbreak. Moreover, it has also been used to predict malaria outbreaks and severe chronic diseases by analyzing the data from previous cases. A prime example of machine learning serving this very purpose is the ProMED-mail [33]. It is an Internet-based reporting platform that monitors diseases- both pre-known and emerging and provides alerts about outbreaks in real-time. Table 2 describes the recent contributions and developments of AI in the healthcare domain. 

## 3. Key Challenges of AI in Healthcare

**Figure 9:**
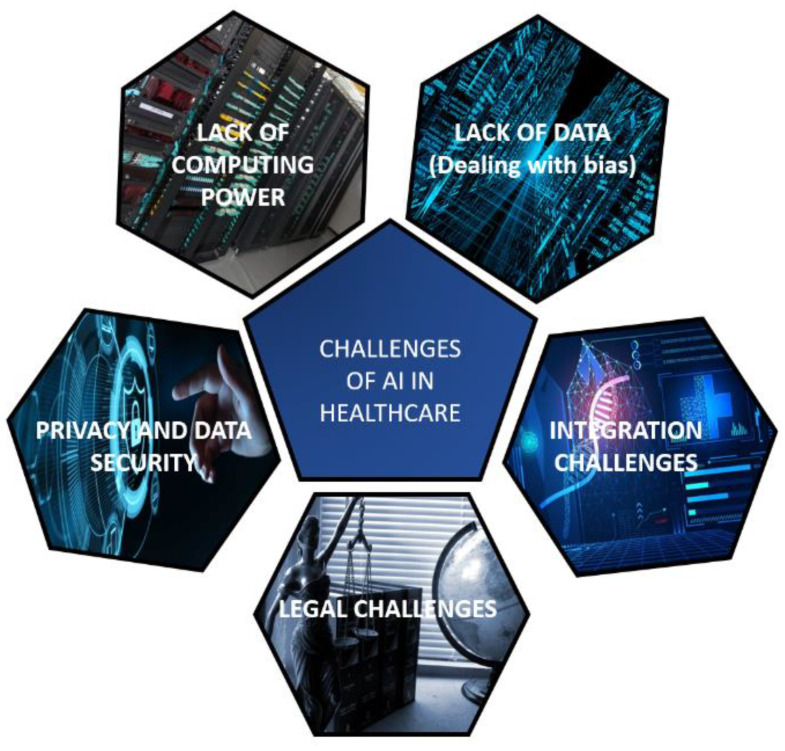
Key Challenges of AI in Healthcare.

Nothing good comes without its own share of difficulties; as such, while it is a given that AI will revolutionize the healthcare industry, one must also be aware of the key challenges that would come up in the case of applying the AI solutions to various healthcare applications. Figure 9 shows the key challenges faced in applying AI to the healthcare industry. While some of these challenges are inherent to any AI-based system, some of them are particular to the field of healthcare and medicine.

### 3.1. Lack of Computing Power

Big data applications require considerable processing power. The bigger the model to be trained, the more is its computational power demand. Healthcare requires larger models to be trained. It needs Neural Networks with multiple layers, and the processing needed in training these models is mostly out of the capabilities of a normal desktop computer. If one tries to train on such devices, then there is a high chance that either the machine will overheat and crash or it will take too many hours or even days to train the models. Thus, a lack of computational power is a very important challenge for integrating artificial intelligence with healthcare. Unfortunately, most hospitals and other healthcare facilities may not often house such powerful systems needed to run these algorithms to the best of their capabilities [40].

### 3.2. Lack of Consolidated Health Data and Dealing with Biases in the Models

In recent times, there has seen a rapid increase in the amount of data available for healthcare and medical diagnosis processes. However, a list of consolidated unbiased datasets is not available for fellow researchers. Moreover, sometimes it is challenging to collect real-time and near-real-time data for various healthcare applications such as detection of dementia/Alzheimer’s, classification of the cardiac condition of patients, stress, obesity, vital signs, patient’s geolocation data for pandemic detection, and a variety of health conditions [41]. In the case of data availability, it is difficult to collect reliable data without any ambiguities and biases. Therefore, having consolidated and verified data is another key challenge in applying AI in the medical field.

### 3.3. Patient Data Security and Privacy

The medical profession gives a lot of importance to maintaining a patient’s confidentiality. However, the moment the data are made available on the cloud and added to datasets for training AI algorithms, there is a high chance of this privacy being compromised and the data being leaked. Therefore, many people are not comfortable with the dangers of data leaking and thus are vary of further integration of AI into healthcare [42].

### 3.4. Challenges of Integrating AI Algorithms into Existing Health Infrastructure

We need to remember that the AI algorithms are not stand-alone solutions; rather, they add to the existing ones so that performance can be further improved. Thus, developing AI algorithms to work as separate programs, which would only add one more step to the existing procedures, will not work as healthcare professionals would not find a very good market. Therefore, care needs to be taken to integrate the algorithms and technologies developed and integrated as seamlessly as possible with the current healthcare frameworks.

### 3.5. Legal Challenges

Leveraging patient data to train the AI models to find the patterns between symptoms and other indicators and the possible diseases requires constant interaction with and feedback from the patients themselves. Furthermore, as mentioned above, medical data have many privacy restrictions, and infringement is dealt with differently in areas under different jurisdictions. Thus, there is a chance for the AI development companies to run afoul of these laws, and care needs to be taken to maintain patient confidentiality while obtaining the data they desire [43]. Table 3 lists the key challenges of AI to the healthcare industry. 

The undertaken study’s scope is limited to the study of the application of AI, particularly machine learning (ML) and deep learning (DL) in the field of immunology and its subdomains. However, some major contributions of the study are listed below:

1. Discussion of the evolution of AI in healthcare, its applications, and the challenges faced in integrating AI into healthcare.
2. The different subdomains and subcategorizations of health diseases have been discussed.
3. Immunology as a domain of healthcare, as well as its subdomains, have been detailed.
4. The different machine learning and deep learning techniques and algorithms available to us currently have also been discussed.
5. A detailed review of recent applications of ML and DL in the immunology domain has been conducted.
6. Finally, research gaps as understood from the survey and possible solutions for the same are proposed.

## 4. Classification of Health Diseases

**Figure 10:**
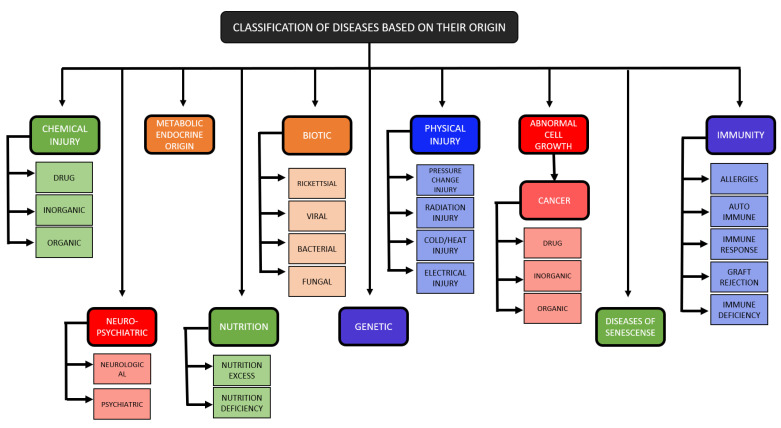
The Representation of Classification of Health Diseases.

A disease is referred to as any type of illness in either humans or other species. A disease can be acute if the infection is small or chronic if the infection period is comparatively large. Health diseases range widely, not only in humans but in all species present worldwide. They can be divided based on various factors like size, biochemical characteristics, interaction manner, etc. In the undertaken study, we have narrowed them down to a broad domain of diseases and eight major domains: topographic, anatomic, physiological, pathological, etiologic, juristic, epidemiological, and statistical [44]. Figure 10 represents a detailed classification of health diseases.

### 4.1. Disease of Genetic Origin

Autoimmunity refers to the system in which an organism’s immune system responds against its healthy cells and tissues. The diseases falling under this system are referred to as autoimmune diseases. Genetic factors, environmental factors, or generally induced autoimmunity can also develop from both the factors combined. Considering genetic factors, diseases of the genetic disorder result from certain mutations in the DNA of chromosomes. Any major or minor alteration can lead to defective synthesis and malfunctioning of proteins. Depending upon the level of mutation, the cases of a genetic disorder are diagnosed concerning fatal conditions. Large mutations are very rare as they generally result in the natural abortion of the fetus before it is born, whereas smaller mutations give birth abnormalities. Most genetic abnormalities such as spina bifida can be detected at birth, whereas abnormalities such as Huntington’s chorea might show symptoms at a very later stage in life [45,46].

### 4.2. Chemical Injury Due to Poison

Autoimmunity can trigger healthy cells to react and produce toxins that further damage the healthy cells and issues of the organism with an autoimmune disease. Apart from genetic and environmental factors, many external factors can result in autoimmunity and lead to severe medical conditions. These factors usually include the entry of drugs or chemicals into the human body. It depends on the concentration of the reaction that determines how effective it can be. Poison with a large concentration will be more toxic than the one with a relatively lower concentration. Each poison has a specific site in the cellular body to attack cells or organs such as the liver or kidneys. Poisonings can be caused either due to the different drugs available in the market or other chemicals used in and around the household. Even common over the counter medications can cause intoxication and poisoning. Furthermore, pesticides, barbiturates, opiates, and even insecticides can lead to poisoning [47,48].

### 4.3. Physical Injury

The injuries such as wounds, cuts, and infections are classified as physical injuries. Depending on the severity of the trauma, the damage can be severe or low. The symptoms and effects of most physical injuries can be seen instantly, whereas a few injuries such as internal bleeding might show late symptoms and, hence, late detection. Alterations can cause physical injuries in temperature, such as frostbite if the temperature is too low and severe burns if the temperature is too high. Electrical injuries can be caused when an electric current passes through the body, which can cause instant death or severe illness depending on the voltage of current and time of contact with the body. High voltage exposures can lead to more long-term complications, longer hospital stays, and surgeries to help the patients recover. Therefore, they have a high mortality rate and far-reaching socio-economic consequences [49]. Radiation injuries can have both positive and negative effects. It is used in cancer treatment to destroy cancer cells, which is positive, but the other healthy cells that can withstand the same energy are negative. The biggest fear factor related to these injuries is that there is no way to figure out their impact until it’s too late when the body has absorbed too much radiation. Then, the effects of the exposure become more pronounced and sometimes even debilitating or life-threatening. Studies indicate that PTSD (Post Traumatic Stress Disorder) can elevate the risk of developing an autoimmune disease. Severe physical injuries and stress disorders can weaken the proper functioning of the immune system [50].

### 4.4. Disease of Senescence

These are the diseases that start to develop with age. Our body matures in the first half of our life span and ages in the second half. Diseases such as atherosclerosis, or fat deposition in the arteries, and arthritis, the disease of joints causing pain and reduced mobility, are examples of diseases of senescence. Senescent cells are produced throughout our lives and sometimes even have positive uses, such as immunity and healing wounds. However, their accumulation with age is sometimes a cause for alarm as they contribute heavily to age-related diseases and even lead to morbidities. With time, the process of ageing defeats a healthy, well-functioning human system. The factors such as chronic inflammation play a major role in the process of ageing. The immune system is directly affected by the same. Therefore, the ageing process might directly help develop an autoimmune disease because of poor cells or might indirectly enhance the risk of developing one by being an initializer for many other diseases [51]. 

### 4.5. Diseases of Immune Origin

An organism’s immune system is present to prevent various kinds of disease-causing agents such as bacteria, fungi, viruses, etc. A disease occurs when either the immune system cannot fight the cause of the disease or if the immune system generates the wrong antigen. In both cases, a dysfunction happens, and disease comes up. Another major cause of mortality in both children as well as adults is autoimmune disorders. They occur when the immune system’s capabilities of distinguishing between self and non-self become compromised. They are caused by both environmental as well as by genetic factors [52].

### 4.6. Diseases of Biotic Origin

All species are infected by biotic agents such as bacteria, viruses, fungi, and other parasites. The agents that can infect and cause the disease are pathogenic, whereas those that cannot cause disease are non-pathogenic. These infections range from the common cold to severe bacterial and fungal infections that might be fatal. These external agents introduce toxins and infections to the body, which can be a major reason for autoimmunity as they hinder the proper functioning of the immune system. 

### 4.7. Diseases of Nutrition

The diseases of nutrition can be caused either by excess or deficiency of important nutrients in the body. The very common disease is obesity, which comes under diseases of nutrition excess, and occurs when a person’s daily intake is too large compared to the required amount. It can cause severe illnesses such as high blood pressure, difficulty in breathing, PCOS (polycystic ovary syndrome) in females, etc. On the other side, malnutrition is a type where a person’s body is deficient in the required nutrients. Anaemia is a very common example of the same and is caused by a lack of Vitamin B12. These diseases can be controlled by balancing the diet and doing regular physical activities to keep the body healthy and safe from diseases. Lack of nutrition might not directly lead to the development of autoimmunity. Still, it can lead to other diseases such as biotic origin, genetic origin, and many more. A lack of nutrition means a weak immune system that can be easily attacked. Therefore, these diseases can further enhance the risk of autoimmunity [53]. 

### 4.8. Diseases of Abnormal Growth of Cells

Human bodies are made up of cells; these cells further, when grouped, make tissues. Therefore, in case of any injury or dysfunction of any part, the growth of cells in that part increases for recovery. This growth of cells is termed hyperplasia. Once healed, the injured part means that the hyperplasia process should stop, but abnormal cell growth occurs in cases that do not happen. In worse conditions, where the cells can no longer respond to the growth inhibitory factors, tumors and cancer is formed. Cancer can be because of genetic reasons, chemicals, or radiations such as UV. These tumors damage the healthy cells; the body is contained with cells that are not healthy and are working against it. Hence disease of abnormal growth of cells is a prominent example of autoimmunity [54].

### 4.9. Diseases of Neuropsychiatric Origin

These diseases are caused by malfunctioning or disruptions in the function of the human nervous system. The nervous system is a very complex structure and the tasks performed need to be very specific and hence very difficult to process. In cases of diseases of neuropsychiatric origin, there is improper or malfunctioning of the neural system. The diseases can either be psychiatric, such as obsessive-compulsive disorder, personality disorder, or neurological diseases like Alzheimer’s and Parkinson’s. [51]. Psychotic disorders and autoimmune diseases both have similar risk factors, majorly infections and stress. Hence, neuropsychiatric and autoimmune diseases can complement each other in many scenarios [55].

### 4.10. Diseases of Metabolic-Endocrine Origin

These diseases are related to the metabolism of the person. They are usually genetic or present by birth. A few can be because of hormones, either by overproduction or by underproduction of hormones by a particular gland in the body. The treatment includes either supplementation or surgery to destroy the gland. These diseases are related to the metabolism of the person. They are usually genetic or present by birth. A few can be because of hormones, either by overproduction or by underproduction of hormones by a particular gland in the body. The treatment includes either supplementation or surgery to destroy the gland. Taking the genetic factors into consideration, autoimmunity is very likely for a human system with metabolic-endocrine diseases [52].

## 5. Immunology and Its Subdomains

**Figure 11:**
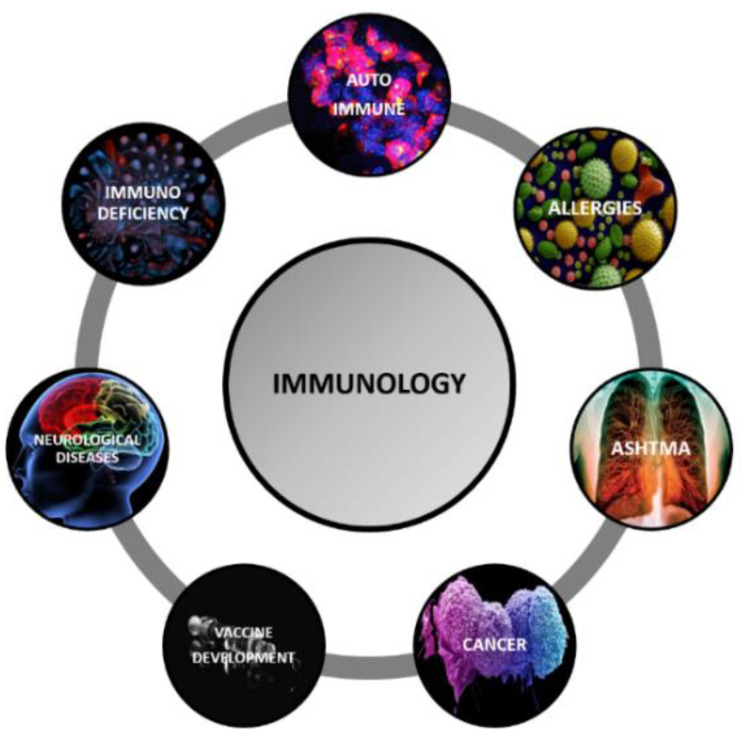
The representation of subdomains of immunology.

The area of application that we have focused on in this paper is AI in immunology. Immunology refers to biomedicine, which deals with the body’s response in particular to external contaminants. The immune system is the body’s line of defence against different kinds of infections. If the immune system does not work properly, it leaves the body susceptible to infections and diseases. Moreover, recent research has shown that diseases that were originally not known to be immunological in origins, such as cancers, metabolic disorders, neurodegenerative disorders like Alzheimer’s and even cardiovascular disorders, also have roots in this field. Thus, immunology is one of the most important and upcoming fields in the area of medicine. Immunologists analyze the body’s responses and the various visualizations of the different diseases such as CT scans, X-rays, etc., to try and identify the immune biomarkers or individual responses that the body has to formulate an action plan to help fight the infections or boost the immune system [56]. Figure 11 represents various subdomains of immunology. There are two main kinds of immune responses that our body possesses: innate and adaptive immunity. Both of these help our immune system to distinguish the external disease-causing pathogens from the self and help in their elimination [57]. Innate immunity is the kind of defence that is generic. The immune system’s first line of defence as its reaction to any pathogens is the same. This response is often enough to battle the pathogens and protect our bodies from infections. In addition, the natural physical barriers that we process, including our skin, saliva, and the hair and mucus in our nose, help supplement our innate immune system. The second type of immunity is called adaptive immunity. As the name suggests, it is an adaptive response generated by the immune system encountering certain pathogens. It is a specifically tailored reaction and is much more effective in destroying the pathogen it counters, thus protecting our bodies. The concept of vaccines is built on top of the idea of adaptive immunity. It involves antibodies and T cells [55,58]. While this is all well and good, there are times and situations where the immune system cannot differentiate between self and pathogens. When this happens, the immune system attacks the body itself, called an autoimmune disorder [39]. Autoimmune disorders can be divided into two categories: the primary autoimmune disorder like diabetes type I, which develops at birth and secondary immune disorders like rheumatoid arthritis, which develop later on in life [54]. Another subfield of immunology is the field of immunodeficiency disorders. It covers the disorders that develop due to a deficiency in the immune system, preventing it from successfully countering that infection. While primary immunodeficiency disorders such as CVID (common variable immunodeficiency) are rare and develop directly from birth, secondary immunodeficiency disorders develop later in life, often due to some infection or exposure and are fairly common, including AIDS (acquired immunodeficiency syndrome), which develops due to HIV (human immunodeficiency virus) infection [58]. The opposite situation to the immune system not working is the immune system over-responding. It means that even if a seemingly harmless pathogen or foreign matter enters the body, the immune system sees it as a threat and reacts too strongly. Such a situation might even lead to outward signs like inflammation, rashes and sneezing and coughing. These allergies can be in response to ordinary things like certain foods, pollen in the air, etc. The inflammation is caused due to the release of chemicals by the immune system cells and is a direct result of an overactive immune system. Asthma can also have an allergen as its source, though not all the time. Asthma is caused when the immune system reacts to inhaled pathogens by thickening the walls of the airways. It makes it difficult to breathe and makes asthma a debilitating and sometimes deadly affliction. Again, the inflammation occurs due to the presence of inflammatory cells in the airways. Furthermore, these then lead to bronchoconstriction along with the secretion of mucus. This inflammation is not easy to control and often can become persistent as well. Cytokines and chemokines play a very important role in the development of asthma and its symptoms [56]. Another important field in immunology is immunotherapy. In particular, cancer immunotherapy. Cancer is a disease characterized by abnormal cell growth, both malignant and benign.

The biggest problem with cancer cell growth is that these cells can avoid destruction by the immune system. Due to this, the immune system needs to be trained and manipulated to fight these cells properly, which is termed immunotherapy. Certain cancers for which research is actively being done include breast cancer, small cell lung cancer, colorectal cancer, skin cancer, etc. Identifying immune biomarkers to judge and direct the immune system’s response is a key area of research for cancer treatment [58]. Lastly, one of the most important applications of immunology is in the development of vaccines. Vaccinations work on the principle of introducing small amounts of weakened pathogens and disease-causing microorganisms into the human body, thus triggering an immune response against them. Since the microbes are already weakened, the body can easily fight off the infection and build immunity by generating the correct antibodies. These antibodies then stay in the body for a while, thus making sure that the immune system is primed to take action the minute the microbes enter our body again. Vaccinations, since their inception, have saved countless lives [59]. COVID-19 is the human race’s biggest bane in the current scenario. The development of vaccines for the same has been and continues to be one of the most important tasks in the medical research area. However, proper research has to be done about the immune system response, immune checkpoints, and biomarkers for the disease to build the vaccine. Furthermore, even mental disorders like depression, anxiety, schizophrenia, functional disorders, Alzheimer’s disease, etc., find a base in immune responses and other kinds of heart and lung diseases. 

### Key Contributions of AI in the Field of Immunology

AI technologies, in particular the classification algorithms in machine learning, can work even at the microscopic levels. They can predict which genotypes and their presence can lead to a poor prognosis [60]. They can even detect the phenotypes and further classify them to predict the chances of occurrence of a particular disease and its severity. This molecular classification helps doctors with charting a treatment plan—one of the most common diseases for which this can be used in HIV [61]. Furthermore, ML algorithms can even determine the genotypes which may lead to resistant phenotypes. This is particularly useful as in the case of resistance, multiple factors determine the resistance level, all of which have their particular weight associated with them. Employing ML algorithms, in this case, considerably speeds up the process as opposed to the traditional statistical analyses. Not only classification algorithms are employed: we can even make use of image-based phenotype detection algorithms. Instead of relying on microscopic analysis done by doctors, which could be prone to human errors, especially in today’s day and age of high volume data generation, these image-based phenotype detection algorithms can not only eliminate chances of error but are also able to study a much wider variety of phenotypes in a much shorter period. These can then be used to determine the kinds of immune responses which would be possible. These include macrophage ac-tiation, lymphocyte infiltration, etc. [62].

Apart from these, immunopeptidome detection is another field of immunology where many highly accurate AI algorithms are often employed. In particular, this field of immunology deals with the development of personalized vaccines. More importantly, beyond predicting the immunopeptidome based on currently available datasets, ML algorithms can now also be trained on data from in vitro studies. This would help doctors determine the presentability of neoantigens in the case of babies. ML algorithms can successfully predict the presentability of epitomes of MHC-I moleculecules and MHC-II molecules, which is a considerably harder task to achieve [63]. AI can even work more on the surface when it comes to immunology, not just at the molecular levels. It can help shortlist candidates for clinical trials or surveys, analyse Electron Health Records Data for hospitals and nursing homes, and even help create more targeted clinical trials. Moreover, facial recognition technologies can be applied to ensure drugs that are to be administered to patients via pills have been swallowed. Furthermore, AI can even help boost the spirits of the patients by acting as smart companions during their times at the hospital and answering generic queries that patients might have. Hence, we can broadly list some of the key areas of applications of AI algorithms in immunology. SVM and K-Nearest Neighbour Algorithms are predictive analyses algorithms that help in cancer prediction. Other algorithms like Similarity Learning can be used to analyse existing data and predict the survival rates of patients depending upon the subtype of cancer. Clustering algorithms help track T-cells receptor repertoire changes, and as explained above, algorithms such as linear regression help predict the peptide presentation by the MHCs (major histocompatibility complex). In contrast, vaccine adjuvants development relied on the Naïve Bayes Algorithm. Decision Trees are algorithms that help clinicians determine the possible responses a patient may have to a particular drug or treatment. Lastly, deep learning algorithms like Neural Networks help with the histopathological image analysis which the physicians need.

**Figure 12:**
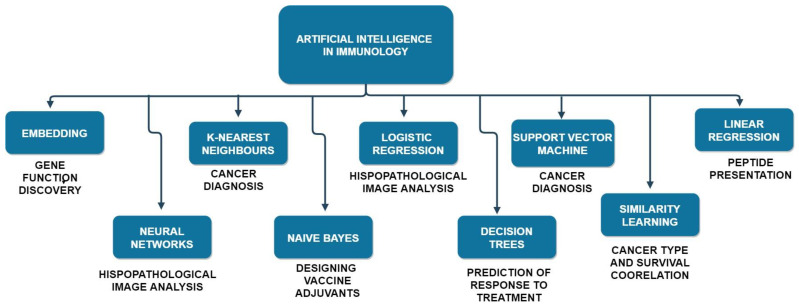
A hierarchical representation of common machine and deep learning methodologies applied to immunology.

Thus, to conclude this section, we can say that the advantage of using AI—ML and DL—to help with the recognition and mapping of biomarkers, checkpoints, inhibitors, and immune responses has made the task of immunologists much simpler. It also reduces the time taken in these tasks exponentially. Furthermore, machines can often find patterns that human beings cannot understand. The accuracy of the predictions also increases when doctors and AI work together rather than either of them alone. Figure 12 represents the common machine and deep learning methodologies applied to the field of immunology.

**Figure 13:**
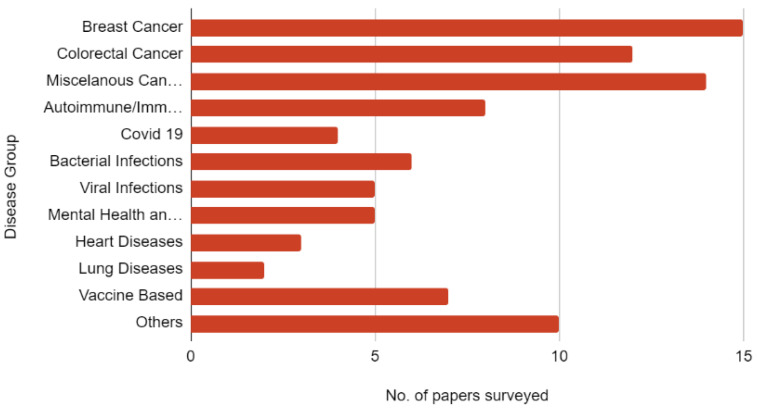
The bar chart representation of the distribution of literature of various immunology subdomains.

Figure 13 shows the number of papers reviewed per subcategory for the undertaken study. Any study conducted using AI requires a dataset to be used. While some researchers prefer to build their dataset, open datasets are a great help in training the models designed by the researchers or even for testing them. Table 4 shows a list of publicly available datasets which can be used in the field of study of the application of AI in immunology.

## 6. Applications of Machine Learning in Immunology

Machine learning is the branch of artificial intelligence that aims at automating the building of analytical models. It is based on the belief that machines can learn from data, identify the patterns between data points, and make decisions with minimal human intervention. There are four broad types of machine learning: supervised learning, semi-supervised learning, unsupervised learning, and reinforcement learning. In addition, another kind of machine learning technique is called evolutionary learning [67,68,69,70,71,72]. 

The kind of data used for training the machine learning model determine the type of ML. There are two types of data—labelled data and unlabelled data. When the model is trained with labelled data and made to predict the values for unlabelled data, it is referred to as supervised learning. During the training stage, supervised learning handholds the machine, showing it what answers are to be expected till it learns the pattern and then predicts the classes on its own for unlabelled data during the testing stage. This way, the model is trained, and still satisfiable high accuracies are achieved [73]. Supervised learning algorithms are further divided into two categories based on the type of output required. In the case of categorical data, the class of machine learning algorithms used are called classification algorithms, and in the case of continuous data, regression algorithms are used [74]. In regression, the first major type of algorithm is linear regression. This algorithm assumes that there is a linear relationship between the input data and the output data. Thus, the output variable(s) can be calculated by a linear combination of the input variables. The decision-making process involves using the values of the linear combination of features to classify similar items [75]. The second and equally well-known regression algorithm is the logistic regression algorithm. This algorithm, as the name suggests, deals mostly with binary classifications. Its basic form is a statistical model that models a binary dependent variable using a logistic function. However, many more complex extensions also exist. 

Decision trees are yet another machine learning model which are highly useful in medical analysis. These algorithms use the tree structure as the basis and can solve regression and classification problems. They have had wide applications in medicine and have a lot of scope for future applications. Although the algorithm has certain shortcomings, it also has checks and procedures to resolve the most problematic situations and deal with their shortcomings, mainly via extensions. These include handling missing data and even understanding and averting the far-reaching consequences of even small changes made in the attribute data. The algorithm also has extensions that help it deal with imprecise information [76]. 

Support Vector Machines are supervised learning methods used for classification, regression, and outlier detection. They use classification algorithms to solve two-group classification problems. Once an SVM model is given a set of labelled training data for each class, it can then classify all the remaining values given to it [76]. Next in the set of supervised learning algorithms is the Naive Bayes Algorithm. It works on the concept of applying the Bayes’ Theorem with the “naive” assumption of conditional independence between every pair of features given the value of the class variable. The algorithm is usually preferred when the output is categorical due to its propensity to produce sometimes inaccurate probability estimates, despite its robustness in identifying the correct class with maximum probabilities. Furthermore, this algorithm can also be applied to numeric predictions, however, with slight variations in its execution. It would require incorporating methods such as the production of model trees and kernel density estimators [77]. 

Another supervised learning algorithm that can solve regression and classification problems is the K- Nearest Neighbours or the KNN algorithm. It works on the ideology expressed as “birds of a feather flock together”. For the algorithm to be successful, it is assumed that similar data points will be close to one another. Thus, the lesser the distance between two points, the more similar they are. The applications of this algorithm are similar to those of the other supervised algorithms mentioned above [78]. One of the biggest challenges of AI in healthcare is the lack of large amounts of labelled data needed by supervised learning algorithms. At the same time, there are an abundance of unlabelled data available. Manually annotating and labelling these data would take loads of human power and be very time-consuming. Herein lies the advantage of semi-supervised learning [79]. It takes huge amounts of unlabelled data and small labelled data. It trains the classifiers to automatically and accurately label the rest of the data, thus reducing time and effort. 

The next category of machine learning algorithms is the unsupervised learning algorithms. These algorithms work with unlabelled data, and it allows the model to run without interfering with letting it discover unknown patterns between the data points. One disadvantage of this machine learning algorithm is that the user has to interpret and label the classes or clusters generated as no labels are made available to the algorithm beforehand [80]. The first kind of unsupervised algorithm is the K Means clustering algorithm. The user selects the number of clusters beforehand. It can either be done randomly or by pre-processing the data and using techniques such as the elbow curve to figure out the optimal number of clusters. It is an iterative algorithm that works on the concept of creating centroids for each of the clusters. These centroids act as the cluster’s heart, and the rest of the data points are sorted depending upon their similarities and differences from the centroid points of each cluster. There are two types of K Means algorithms: (i) the Agglomerative clustering algorithm and (ii) the Dendrogram clustering algorithm. Agglomerative clustering starts by defining each element as a separate cluster. It then reduces the number of clusters in each iteration by the merging process. An agglomerative fuzzy K-means further includes a penalty term with the objective function to ensure that the sensitivity to the initial cluster centres is reduced [81]. In the case of dendrogram clustering, a possible cluster is represented by each level. The height of the dendrogram represents the level of similarity between two join clusters. 

Another unsupervised clustering algorithm is the DBSCAN algorithm. It is a density-based clustering algorithm whose major advantage over the K Means algorithm is that, unlike k means, one need not define the number of clusters to use the DBSCAN algorithm. The only thing needed is a function that can calculate the distance between the different data values along with a little guidance about how close is considered “close” [82]. The fourth and final branch of machine learning is called reinforcement learning. It focuses on taking a suitable action that will then read to a maximized “reward” in any particular situation. In the case of reinforcement learning, the “correct answers” are not provided to the machine; rather, it decides what to do to perform a particular task as it learns from experience [83]. Table 5 provides a detailed study of applications of machine learning in the various subdomains of immunology. It includes information pertaining to the disease, methodology and sub methodology used, the evaluation metrics applied, and a summary of the work done.

## 7. Applications of Deep Learning in Immunology

A subcategory of machine learning is called deep learning. DL focuses on neural network models. It is mainly used to solve perceptual problems. Another major factor about these algorithms is that previous knowledge has no impact on a recent decision which only depends upon the extracted feature [106,107,108,109,110]. As the name suggests, neural networks are algorithms that try to mimic the functioning of the neurons inside a human brain to recognise patterns and relationships between the different data points. Deep learning algorithms are different. Unlike other machine learning techniques, DL algorithms (neural networks) keep improving their performance with large data. Other machine learning algorithms instead reach a plateau, after which increasing the amount of data does not improve the algorithm’s performance. These algorithms are becoming increasingly more popular in healthcare. In this section, let us look at some DL algorithms and their application in the immunology domain. 

The first type of Neural Network which we will be discussing is the Convolutional Neural Network. It is also called the ConvNet. In this section, let us look at some DL algorithms and their application in the immunology domain. It works on the concept of taking an image input and then assigning weights and biases to the different objects within the image and then finally learns how to differentiate them from one another. Thus, it requires relatively lesser pre-processing. It is different from all other classifiers as it can pick out the subtle sophistication and details within an image. It does this by capturing the temporal and spatial dependencies within an image by applying the required filters. These CNNs are applied in medical image categorisation using either trained or self-trained CNN models or unsupervised pre-trained models [111]. 

The second type of DL algorithm to be considered is the Recurrent Neural Network or RNNs, particularly the LSTM algorithm. The basic differentiation factor of recurrent networks is that they have loops. It means that they can remember information for a certain duration of time. LSTM stands for Long Short Term Memory Networks. LSTMs, unlike the other neural network algorithms, are capable of remembering long term. It performs the following tasks: It decides what data to keep and discard, decides what other data are to be added to each particular cell, and the final output [112]. 

Multi-Layer Perceptron or MLPs are the next algorithms in the list. As the name suggests, an MLP classifier is a neural network model. In particular, it is a feed-forward neural network that uses backpropagation for training the network. It consists of several layers of input nodes which form a directed graph between the input and output layers. 

Another set of algorithms similar to the Convolutional Neural Networks is the Generative Adversarial Networks or GANs. Like CNN’s, the purpose of a GANS algorithm is to identify the patterns between the different data values given and generate more samples that could have been taken from the original dataset. The training of the GANS generative model is done by taking the problem as a supervised learning problem and making two sub-models within it. The generator model will generate the new examples and the discriminative model that will classify the examples as Real or Not Real. The model training, which is carried out negatively, is complete when the discriminator is fooled about half the time [113]. 

Deep Belief Networks are another kind of algorithm amalgamating statistics and probability with neural networks and machine learning. Their main aim is to classify the data into different categories. It is achieved by creating a structure with multiple layers wherein there is a relationship between the layers rather than the values. An example of a kind of DBN is a Restricted Boltzmann Machine. It is an unsupervised network kind algorithm that contains multiple layers such that the invisible layer of one subnetwork is the visible layer of the next [114]. Table 6 provides a detailed study of machine learning applications in the various subdomains of immunology, as discussed above. It includes information pertaining to the disease, methodology and sub methodology used, the evaluation metrics applied, and a summary of the work done.

## 8. Open Research Issues; Research Challenges; and Future Directions

**Figure 14:**
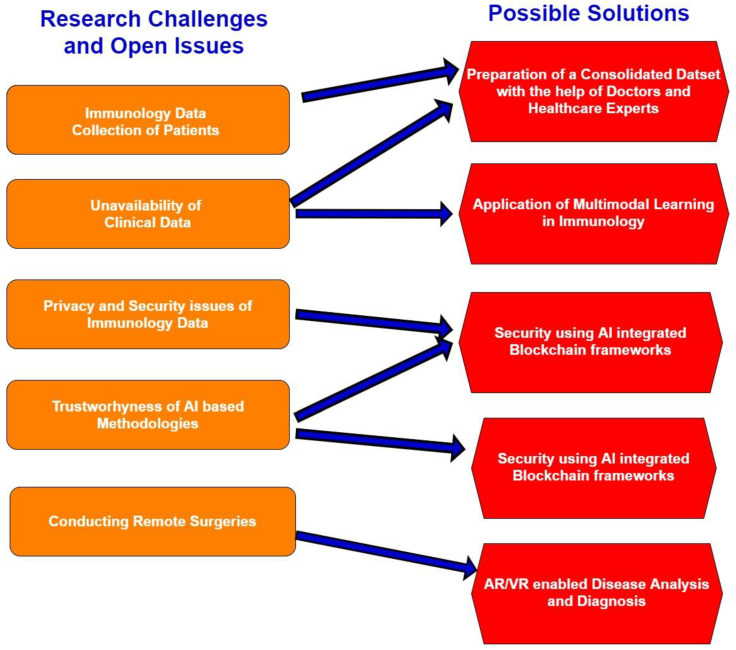
A logical mapping of Research Challenges, Open Research Issues, and Possible Solutions.

This section has discussed numerous open research issues, research challenges, and future directions for fellow researchers. Figure 14 represents a logical mapping of various research challenges, open research issues, and possible solutions.

### 8.1. Preparation of a Consolidated Dataset

In recent times, there have been numerous researches were carried out by fellow researchers. However still, a consolidated dataset that consists of immunological data and clinical findings are not available. Furthermore, it is a fact that the government and private hospitals are reluctant to provide confidential data due to security and privacy-related issues related to patient history. 

### 8.2. Application of Multimodal Learning in Immunology

Fellow researchers have conducted a few research works in immunology by applying machine and deep learning methodologies such as detecting bacterial infections in the current pandemics, medical imaging, viral infections, cardiac arrest conditions, diabetes, etc. In recent times, there have been numerous researches were carried out by fellow researchers. However, the application of multimodal learning in detecting infectious and vector-borne diseases has remained open research problems due to the unavailability of clinical data, data fusing challenges, and additional computation power requirements [136].

### 8.3. Securing Immunological Data Using AI Integrated Blockchain Frameworks

Blockchain-based security can secure electronics record management (EMR) systems using a secure hashing algorithm such as SHA-256 [125,137]. However, to make better machine learning-based decisions, it is essential to integrate Blockchain-based security can secure electronics record management (EMR) systems using a secure hashing algorithm such as SHA-256. However, to make better machine learning-based decisions, it is essential to integrate AI and Blockchain technology to make accurate decisions with trustworthiness and transparency.

### 8.4. Targeted Healthcare Using Explainable and Interpretable AI

In targeted healthcare applications, the usage of explainable and interpretable AI methodologies is minimal due to the complexity of the design and development of explainable AI and interpretable AI integrated systems. The explainable AI integrated healthcare systems can perform patient history analysis and medical diagnosis and explain the critical healthcare conditions to healthcare experts and caretakers to make critical healthcare decisions. 

### 8.5. Augmented Reality/Virtual Reality (AR/VR) Enabled Immunology Disease Analysis

The technologies such as AR/VR are at the initial stage of research. However, they can bring innumerable to design AR/VR enabled healthcare ecosystem, remote healthcare monitoring and diagnosis, and remote surgeries. However, it is challenging to integrate AR/VR technologies with AI, IoT-enabled healthcare systems and security and privacy technologies such as Blockchain.

## 9. Conclusions

Due to the advent of technologies such as AIoMT (Artificial Intelligence of Medical Things), genetic intelligence algorithms and approaches, and smart immunological methodologies, fellow researchers have conducted numerous AI-related research works in healthcare to detect various types of diseases such as autoimmune diseases, immunological deficiency syndromes and disorders, inflammatory diseases, lymphoproliferative disorders, etc. In addition, immunology research is carried out on diseases such as cancer, mental health, bacterial infection, COVID-19, and many more. Furthermore, to understand immunology related health risks, it is essential to understand various types of cells, their functionalities, membrane molecules, and monoclonal antibodies. In this study, we have emphasized discussing the evolution of AI in healthcare, represented detailed process flows of various health domains such as medical image diagnosis, drug discovery and manufacturing, personalized medicine, clinical trials and data collection, smart records management, etc. In the initial section, we have completed a detailed discussion of aspects of AI in healthcare, various health domains using detailed process flow representations, key challenges, and recent developments in AI in healthcare. The intermediate part of the study is focused on carried out discussions of health disease classifications with the main focus on immunology. Furthermore, we have also presented a detailed review work of machine learning and deep learning methodologies applied to solve immunology related health problems. In the final section, we have discussed the future research directions and alternative solutions in immunology.

## Figures and Tables

**Table 1:** List of Terminologies and Abbreviations.

Terminology	Description
SL	Supervised Learning
UL	Unsupervised Learning
RNN	Recurrent Neural Network
SVM	Support Vector Machine
KNN	K-Nearest Neighbours
DBSCAN	Density-Based Spatial Clustering of Applications with Noise
CNN	Convolutional Neural Network
LSTM	Long Short-Term Memory
MLP	Multi-Layer Perceptron
GANs	Generative Adversarial Networks
DBN	Deep Belief Network

**Table 2:** Recent Contributions and Developments in AI in Healthcare.

Authors	Year	Contribution
Guoguang Rong et al. [33]	2020	The paper focuses on AI developments in disease diagnostics and prediction, living assistance, biomedicine, biomedical research, etc. The major area covered by the reviewers is biomedicine.
Silvana Secinaro et al. [34]	2021	The review focuses on health services management, predictive medicine, patient data and diagnostics, and clinical decision-making. It gives an overview of how AI is being used in these areas and briefs about the developments that need to be carried out.
Thomas Davenport et al. [35]	2019	This review paper showcases how AI is being used in healthcare, the relevance between AI and healthcare, various applications, and the implications related to the same.
Pouyan Esmaeilzadeh et al. [36]	2020	This study examines AI medical devices’ perceived benefits and risks with clinical decision support (CDS) features from consumers’ perspectives, sheds more light on factors affecting perceived risks, and proposes recommendations to practically reduce these concerns.
Jonathan Waring et al. [37]	2020	A state of the art review of 101 papers identifies the potential opportunities and barriers to using AutoML in healthcare and the existing applications of AutoML in healthcare.
Onus Asan et al. [38]	2019	This paper shows the clinician’s point of view: how AI is helping their work and domain, the challenges that usually arise, and the possible future scope of AI in healthcare.
Jiamin Yin et al. [39]	2021	Fifty-one healthcare studies were reviewed, targeting clinical tasks, disease diagnosis, risk analysis, and treatment.
DonHee Lee et al. [20]	2021	Reviews the current state of artificial intelligence [AI]-based technology applications and their impact on the healthcare industry, the details of those opportunities and challenges to provide a balanced view of the value of AI applications in healthcare. It is clear that rapid advances in AI and related technologies will help care providers create new value for their patients and improve the efficiency of their operational processes.
Adam Bohr et al. [21]	2020	Applications that are directly associated with healthcare and those in the healthcare value chain such as drug development and ambient assisted living are discussed in this review.
Nagendra et al. [22]	2020	This review compares the performance of diagnostic deep learning algorithms for medical imaging with that of expert clinicians.

**Table 3:** A list of Key Challenges in AI in Healthcare.

Key Reference	Year	Research Challenges	Discussion
Ghayvat et al. [23]	2021	Integration, Legal, Data collection	− Integrate AI into existing workflow − Requires Consolidated Data − Application of AI in healthcare (it is challenging to apply AI models into healthcare) − Legal Challenges (concerning data sharing agreement)
Kelly et al. [42]	2019	Integration, Data Veracity	− Retrospective versus prospective studies − Peer-reviewed randomized controlled trials as an evidence gold standard − Metrics often do not reflect clinical applicability − Difficulty comparing different algorithms − Challenges related to machine learning science − Dataset shift − Accidentally fitting confounders versus true signal − Challenges in generalization to new populations and settings − Algorithmic bias − Susceptibility to adversarial attack or manipulation − Logistical difficulties in implementing AI systems − Achieving robust regulation and rigorous quality control − Human barriers to AI adoption in healthcare − Algorithmic interpretability is at an early stage but rapidly advancing − Developing a better understanding of the interaction between human and algorithm
Flint et al. [43]		Accuracy	− In-Patient Mobility Monitoring and Clinical Trials for Drug Development − Quality of Electronic Health Records (HER) − Industry Challenges Persist

**Table 4:** A list of datasets applied to immunology.

Dataset	Year	Nature of Data	Public	Labelled	Balanced	Updating
Visible Human Project [64]	1995	Supervised	√	√	√	×
Phil Image Data [53]	2018	Supervised	√	√	√	×
Clinical Questions Collection [58]	2003	Supervised	√	√	×	×
NLM Meeting Abstracts Data [55]	2010	Supervised	√	√	√	×
CCRIS Database [54]	2011	Supervised	√	√	√	×
ChemlDplus [56]	2007	Supervised	√	√	×	√
GENE-TOX [57]	1998	Supervised	√	√	√	×
Hazardous Substances Data Bank (HSDB) [59]	2021	Supervised	√	√	×	×
LactMed Database [65]	2006	Supervised	√	√	√	×
TOXLINE [66]	2006	Supervised	√	√	√	×

**Table 5:** A Review of Applications of Machine Learning in Immunology.

Authors	Disease	Methodology	Sub Methodology	Evaluation Metrics	Summary
Andrew J. Sweatt et al. [84]	Pulmonary Arterial Hypertension	Machine Learning	Unsupervised Learning (Clustering algorithm)	Gaussian graphical modelling	Classification of PAH Concerning 4 clusters With the help of clustering algorithm
Sidhartha Chaudhury et al. [76]	effects of adjuvant formulation on human vaccine-induced immunity	Machine Learning	Unsupervised Learning (Hierarchical Clustering)	Evaluation is done on the basis of difference in the responses concerning truth table	Clustering on the samples concerning the effect of effects of adjuvant formulation on human vaccine-induced immunity
Laura Andrés-Rodríguez et al. [77]	Fibromyalgia (FM)	Machine Learning + Deep Learning	Logistic Regression and NN	Sensitivity	Machine learning and deep learning models was created to classify the value of FM
Jingjing Zhang et al. [78]	bacterial infections	Machine Learning + Deep Learning	Supervised Learning (SVM, NN)	1—Specificity, AUC	Machine learning and deep the learning model is used to classify whether the patients have infection or not
Sidhartha Chaudhury et al. [79]	immune signature of adjuvant formulations in vaccines	Machine Learning	Unsupervised Learning (Hierarchical Clustering)	Evaluation is done on the basis of difference in the responses concerning truth table	Machine learning is used to predict the clusters on the basis of vaccine and body parts
Matthew T. Patrick et al. [80]	Cutaneous Diseases	Machine Learning	Supervised Learning (Classification algorithm)	Precision, Recall, F1-Score	Machine learning classification the algorithm is used for drug repurposing in immune-mediated cutaneous diseases using a Word-Embedding
Jorge M. Arevalillo et al. [81]	Shigella infection	Machine Learning	Supervised Learning (Classification algorithm)	*p*-value and various other parameters	In machine learning the classification algorithm is used to predict the immunity with respect to protection of shigella infection.
Maurizio Polano et al. [82]	Immune checkpoint inhibitors in cancer	Machine Learning	Supervised Learning (Classification algorithm)	ACC [CI] ACC Test MCC [CI] MCC Test	Machine learning method is used to predict responsive immunity conducted in pan-cancer experiment
Noëmi Rebecca Meier et al. [83]	Tuberculosis	Machine Learning	Supervised Learning (Classification algorithm)	ROC-AUC Curve	Machine learning is used to predict the immune responses and classify the Mycobacterium tuberculosis antigens for diagnosis of tuberculosis.
Buranee Kanchanatawan1 et al. [85]	schizophrenia	Machine Learning + Deep Learning	ANN + Supervised Learning	Accuracy, *p*-value and many more	An ANN approach is used to predict the complex association between the neurone in the immunity for quality life in schizophrenia
Juha P. Väyrynen et al. [86]	Colorectal Cancer	Machine Learning	Supervised Learning (Classification algorithm)	*p*-value, AUC	Machine learning algorithms is used to classify the colorectal cancer with the the help of immune cell populations.
Victor Greiff et al. [87]	N/A	Machine Learning	Supervised Learning (Classification algorithm)	Accuracy	Machine learning algorithm for classification is used to predict the development of antibody. Except that the analysis is also focused with sequencing.
Lana G. Tennenhouse et al. [88]	Depression and anxiety (for people suffering from immune-mediated inflammatory diseases)	Machine Learning + Deep Learning	Logistic Regression, Random Forests, Neural Networks	AUC, Sensitivity, Specificity and corresponding 95% CIs	Machine learning and statistical algorithms were used to identify the PROM items that could predict MDD and anxiety disorders with high accuracy. These were assessed via a semi-structured psychiatric interview conducted for a portion of the IMID population.
Hasan AbbasQazmooza et al. [89]	angina, increased atherogenicity and insulin resistance	Machine Learning	Logistic Regression	ROC Curve.	Machine learning algorithm is used for classifying unstable, increased atherogenicity and insulin resistance
Hassan M. Rostam et al. [90]	immune response (macrophage)	Machine Learning	Supervised Learning (Classification algorithm)	*p*-value	Machine learning approach is used to classify the level of disease with respect to images
Hiroki Konishi et al. [91]	Tumor	Machine Learning	Supervised Learning (Classification algorithm)	ROC and AUC	The aim is to this study was to explore the possibility of discriminating BCRs/Igs in tumor and in normal tissues, by capturing these differences using supervised machine learning methods applied to RNA sequences of BCRs/Igs.
Tathiane M.Malta et al. [84]	Cancer	Machine Learning	One class Logistic Regression	Correlation	OCLR was used to identify a set of novel stemness indices in the case of cancer. It was used to identify features based on non-transformed pluripotent stem cells and their differentiated progeny and also to identify till now unknown biological mechanisms involved in the dedifferentiated oncogenic state
En-hui Ren et al. [92]	Ewing sarcoma	Machine Learning	univariate and multivariate iterative Lasso Cox regression	Correlation	Cox regression was used to create an optimal signature which can be used for the determination of ES patient prognosis and is based on the immune-related gene
George A Robinson et al. [93]	juvenile-onset systemic lupus erythematosus				
Adriana Tomic et al. [94]	Influenza Vaccine Responses	machine Learning	Supervised Learning (Classification algorithm)	Confusion matrix	Machine learning classification algorithms are used to predict the labels for vaccine responses
Liang Xue et al. [95]	Lung Adenocarcinoma	Machine Learning	Statistical analysis	Accuracy	Statistical analysis is done on LAUD dataset
Ahmed Mekki et al. [96]	Cancer	Machine Learning	Supervised Learning (Classification algorithm)	AUC, *p*-value,	A machine learning-based approach is used for classification in autoimmune hypophysis in patients.
Naoya Nezu et al. [95]	Intraocular Disease	Machine Learning	Supervised Learning (Classification algorithm)	Precision, Recall, Accuracy, F1 score	A machine learning approach is used to classify labels from Intraocular Disease.
Akira Ono Yukihiro Terada et al. [97]	Lung cancer	Machine Learning	Supervised Learning	Multivariant, *p*-value	Machine learning approach is used for lung cancer concerning immunity
Shayantan Banerjee et al. [98]	sepsis	Machine Learning	Supervised Learning (Classification algorithm)	sensitivity, specificity, false-positive rate, MCC	In machine learning, a classification approach is used to classify complicated species course and mortality rate with respect to 20 genes of immunity in blood.
Bo Peng et al. [99]	pneumonia	Machine Learning	Supervised Learning (Classification algorithm)	ROC, AUC curve	A machine learning, classification based approach is used to classify immune association, pneumonia
Ahmad Y. Abuhelwa et al. [100]	Urothelial Cancer	Machine Learning	GBM	Kaplan–Meier	A machine learning approach is used to solve the survival the outcome with immune checkpoint inhibitors. The disease is urothelial cancer
Gu-Wei Ji et al. [101]	Biliary Tract Cancer	Machine Learning	Clustering	*p*-value	A machine learning approach is used to predict oncologic outcomes for biliary tract cancer
Maximilian Wübbolding et al. [102]	HBeAg-Negative CHB	Machine learning	Supervised Learning (Classification algorithm)	sensitivity, specificity, *p*-value	A machine learning approach is used for early virological relapse after stopping nucleos(t)ide analogues in HBeAg-Negative CHB
Sara Poletti et al. [103]	bipolar and unipolar depression	Machine Learning	Supervised Learning (Regression algorithm)	*p*-value, *t*-value	A machine learning approach is used to predict HC and BD and many other parameters in depression.
J.S. Hooiveld-Noeken et al. [104]	skin cancers	Machine Learning/Deep Learning	ANN	Accuracy	An artificial neural network is used to classify whether the person is responder or not
Awais et al. [105]	Prediction of teeth, skin, and cavity cancer	Machine Learning	Supervised Learning (Regression algorithm)	*p*-value	A machine learning approach is used to predict cavity cancer.

**Table 6:** A Review of Applications of Deep Learning in Immunology.

Authors	Disease	Methodology	Sub Methodology	Evaluation Metrics	Summary
Kamil Wnuk et al. [115]	Tumor	Deep Learning	CNN	HR, Log-rank P	A deep learning approach is used to predict tumors using DNA and immune activity.
Jingcheng Wu et al. [116]	Neoantigen	Deep Learning	RNN	Fivefold cross-validation	A deep learning approach is used for the prediction of neoantigen with the help of HLA-peptide binding and immunogenicity.
Lilija Aprupe et al. [117]	Lung cancer	Deep learning	Deep CNN	Confusion matrix	A deep learning approach is used to classify the labels of lung cancer on the basis of immune cells in lungs.
Leeat Keren et al. [118]	Breast cancer	Deep learning	Neural network	Sensitivity, specificity	A deep learning approach is used to classify breast cancer based on immune cell images.
Michael Widrich et al. [119]	N/A	NLP	Attention model	AUC	An attention-based model is used to predict the labels concerning immune repertoire.
Guangyuan Li et al. [120]	Dengue virus, cancer neoantigen and SARS-Cov-2	Deep Learning	Classification	sensitivity, ten-fold cross-validation	Presented DeepImmuno-CNN model outperformed another prediction workflow when applied to diverse real-world immunogenic antigen datasets, including cancer and COVID-19 infection.
Han, Y et al. [121]	Lung adenocarcinoma	Machine Learning & Deep Learning	naive Bayes, random forest, support vector machine, and neural network-based deep learning	F1 Score, Confusion matrix	Optimized model for personalized management of early-stage LUAD patients.
Zhu et al. [122]	Ovarian Cancer	Deep Learning	mask-R-CNN (MRCNN)	leave-one-out cross-validation	Novel analytic and modelling pipeline of IMC images using deep learning and applied it to predict patient survival rates using IMC data generated from patient samples of treatment-naïve HGSC tumor tissues.
Meng Jiaa et al. [123]	Thyroid Cancer	Machine Learning	Supervised Learning (Classification algorithm)	ROC, AUC	A machine learning approach is used to classify thyroid cancer based on immune infiltration
Zi-zhuo Li et al. [124]	LGG	Deep Learning	Neural network	Confusion matrix	A neural network is used to classify LGG patients based on immunity.
Shaista Hussain et al. [125]	N/A	Deep Learning	Transfer learning	Ground truth	A transfer learning analysis is done for drug anomaly detection.
Sebastian Klein et al. [126]	Tumor	Deep Learning	CNN	AUC	A deep learning approach is used to predict the tumor infiltrating lymphocyte clusters
Ofer Isakov et al. [127]	Inflammatory bowel diseases (IBDs)	Machine Learning	Random forest, simply, xgbTree and glmnet	AUC	A machine learning method was created, which differentiated IBD-risk genes from non-IBD genes using information from expression data and many gene annotations.
When Ning et al. [128]	Periodontitis	Deep Learning and Machine Learning	K-means clustering and ANOVA, support vector machine	cross-validation (CV), accuracy, and area under the curve (AUC)	A deep learning based Autoencoder was applied to identify immune subtypes and key immunosuppression genes. Key factors for the mediation of immune suppression in periodontitis were also identified.
Panwen Tian, Bingxi He et al. [129]	non-small cell lung cancer	Deep Learning	deep convolutional neural network	receiver operating characteristic curve (ROC), Kaplan-Meier curves and Log-rank test	A deep CNN model was created to work with CT images to assess the levels of PD-L1 in a non-small cell Lung Cancer. Furthermore, the response to immunotherapy was also predicted
Carlo Augusto Mallio et al. [130]	COVID-19	Deep Learning	deep convolutional neural network	sensitivity, specificity, AUC, ROC and Mann–Whitney U test	A deep CNN model was applied to CT images of Pneumonia, COVID-19 and ICI pneumonitis to differentiate between the three.
Riku Terrki et al. [131]	Breast Cancer	Deep Learning	convolutional neural network	F-score, an area under receiver operating characteristics curve (AUC), and with accuracy, sensitivity, specificity, precision, pairwise Pearson’s linear (two-tailed) correlation coefficient (r), 3-fold cross-validation and leave-one-out cross-validation	A CNN model was proposed and evaluated based on the antibody-guided annotation to identify and quantify the areas with high immune cell concentration in the case of Breast Cancer using samples stained in haematoxylin and eosin (H&E)
Changhee Park et al. [132]	lung adenocarcinoma	Deep Learning	Supervised Learning (Classification algorithm)	P-value, rho, ROC AUC.	A deep learning approach is used to classify lung adenocarcinoma using LAUD dataset
Chunyu Huang et al. [133]	Pregnancy Outcomes	Deep Learning	Supervised Learning (Classification algorithm)	Accuracy, specificity, Sensitivity	A deep learning approach is used to classify the pregnancy outputs
Xiwei Huang et al. [134]	WBC Counting	Deep Learning	Resnet-50 Neural network	Precision, Recall, and F1_Score	a label-free three-type WBC classification method using the transfer learning technique based on the Resnet-50 neural network.
Priya Lakshmi Narayanan et al. [135]	Ductal carcinoma in situ	Deep Learning	Resnet 101-based RCNN network15, UNet16, MicroNet17	Accuracy (F1_Score), cross-validation	A deep computational framework to [1] to develop and validate a computational pipeline that accurately detects and segments individual DCIS ducts; [2] to characterise the immune microecology for each DCIS duct using spatial statistics on H&E and IHC for TILs; [3] to test the difference in DCIS microecology between samples with pure DCIS and DCIS samples derived from IDC patients (adjacent DCIS, as a surrogate for poor prognosis DCIS).

## Data Availability

Data will be made available based on request.
